# Multiple functions of flagellar motility and chemotaxis in bacterial physiology

**DOI:** 10.1093/femsre/fuab038

**Published:** 2021-07-06

**Authors:** Remy Colin, Bin Ni, Leanid Laganenka, Victor Sourjik

**Affiliations:** Max Planck Institute for Terrestrial Microbiology & Center for Synthetic Microbiology (SYNMIKRO), Karl-von-Frisch Strasse 16, Marburg D-35043, Germany; Max Planck Institute for Terrestrial Microbiology & Center for Synthetic Microbiology (SYNMIKRO), Karl-von-Frisch Strasse 16, Marburg D-35043, Germany; College of Resources and Environmental Science, National Academy of Agriculture Green Development, China Agricultural University, Yuanmingyuan Xilu No. 2, Beijing 100193, China; Institute of Microbiology, D-BIOL, ETH Zürich, Vladimir-Prelog-Weg 4, Zürich 8093, Switzerland; Max Planck Institute for Terrestrial Microbiology & Center for Synthetic Microbiology (SYNMIKRO), Karl-von-Frisch Strasse 16, Marburg D-35043, Germany

**Keywords:** chemotaxis, motility, *Escherichia coli*, environmental adaptation, physiology

## Abstract

Most swimming bacteria are capable of following gradients of nutrients, signaling molecules and other environmental factors that affect bacterial physiology. This tactic behavior became one of the most-studied model systems for signal transduction and quantitative biology, and underlying molecular mechanisms are well characterized in *Escherichia coli* and several other model bacteria. In this review, we focus primarily on less understood aspect of bacterial chemotaxis, namely its physiological relevance for individual bacterial cells and for bacterial populations. As evident from multiple recent studies, even for the same bacterial species flagellar motility and chemotaxis might serve multiple roles, depending on the physiological and environmental conditions. Among these, finding sources of nutrients and more generally locating niches that are optimal for growth appear to be one of the major functions of bacterial chemotaxis, which could explain many chemoeffector preferences as well as flagellar gene regulation. Chemotaxis might also generally enhance efficiency of environmental colonization by motile bacteria, which involves intricate interplay between individual and collective behaviors and trade-offs between growth and motility. Finally, motility and chemotaxis play multiple roles in collective behaviors of bacteria including swarming, biofilm formation and autoaggregation, as well as in their interactions with animal and plant hosts.

## Teaser

This review summarizes the recent advances in understanding the impact of flagellar motility and chemotaxis on various behaviors of bacteria, from nutrient acquisition and population range expansion to interactions among bacteria and with their animal and plant hosts.

## INTRODUCTION

Swimming bacteria are able to monitor changes in environmental conditions as they move and to adapt their swimming pattern accordingly, in order to swim towards their preferred environment. Such biased movement in chemical gradients, called chemotaxis, is one of the longest and most thoroughly studied bacterial behavioral responses. Understanding of the molecular mechanisms controlling the chemotactic behavior has become highly refined over the years, especially in the model organism *Escherichia coli* (Wadhams and Armitage 2004; Colin and Sourjik 2017; Bi and Sourjik 2018). As a consequence, the typical behavior of a bacterial cell in a simple gradient and the underlying biochemistry and biophysics are well understood, and they could be mathematically modelled down to minute quantitative details (Tu 2013; Micali and Endres 2016; Colin and Sourjik 2017; Waite, Frankel and Emonet 2018; Wong-Ng, Celani and Vergassola 2018).

**Figure 1.**
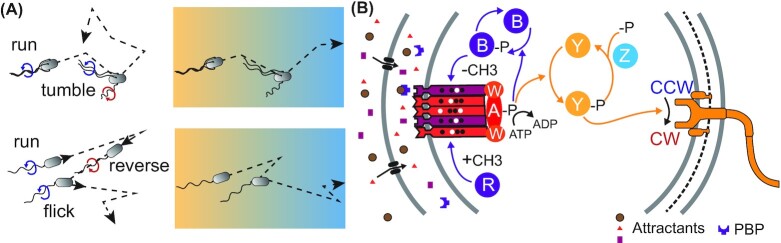
Chemotactic behavior and signaling pathway. **(A)**, Two prominent types of bacterial flagellar motility patterns, run-tumble and run-reverse-flick swimming. Both types of swimming lead to effective diffusion in homogeneous environments and get biased by the chemotaxis pathway to climb up physicochemical gradients. **(B)**, Schematic representation of the chemotaxis pathway of *E. coli*, featuring clustered chemosensory complexes formed by receptors bound to histidine kinase CheA and adaptor protein CheW. Chemoreceptors detect chemical ligands, either directly via their ligand binding domains or indirectly upon interactions with periplasmic binding proteins (PBPs), and modulate activity of CheA. The signal is transmitted to flagellar motor by phosphorylation of the diffusible response regulator CheY, which modulates the direction of motor rotation. The signal is terminated by the phosphatase CheZ. Receptor methylation enzymes, the methyltransferase CheR and the methylesterase CheB carry out adaptation to steady stimulation and provide short-term memory for temporal concentration comparisons.

Flagellated bacteria typically swim in a series of more or less straight runs interrupted by short reorientations (Fig. 1A). In peritrichous bacteria like *E. coli*, runs occur when all flagella rotate unidirectionally (counterclockwise in the case of *E. coli*) and form a bundle that propels the cell body forward (Berg 1975; Macnab 1977). Tumbles, which result from transient reversal of the rotary direction of flagellar motors, cause the flagellar bundle to fall apart and lead to reorientation of the cell body. The strategies of reorientation in polarly flagellated bacteria are more complex and diverse, with several distinct cell motility states, which might have been evolutionary selected to match the respective bacterial habitat (Altindal, Xie and Wu 2011; Constantino *et al*. 2018; Grognot and Taute 2021; Stocker 2011; Taktikos, Stark and Zaburdaev 2013; Xie *et al*. 2011). Regardless of the specific mechanism of reorientation, such run-reorientation behavior results over long times in an active diffusion that enables bacteria to efficiently spread in their environment.

The chemotaxis system modulates the duration of the runs according to perceived changes in environmental conditions, making them longer or shorter, if conditions get better or worse, respectively, to bias the average cell motion towards favorable environments (Berg and Brown 1972; Larsen *et al*. 1974; Macnab and Koshland 1972). The signaling pathway controlling this behavior is highly conserved among bacteria and even archaea (Fig. 1B). Nevertheless, several distinct classes of chemotaxis pathways could be distinguished based on their detailed molecular composition and evolutionary relatedness, some of which control behaviors other than flagellar motility (Gumerov, Andrianova and Zhulin 2021; Wuichet and Zhulin 2010). In contrast to many other bacteria, the genome of *E. coli* encodes only a single motility and chemotaxis system, which moreover functions with a nearly minimal set of chemotaxis proteins. Such comparative simplicity turned *E. coli* into a preferred model for studying signal transduction in bacterial chemotaxis (Bi and Sourjik 2018; Parkinson, Hazelbauer and Falke 2015).

In general, bacterial chemotaxis pathways consist of two modules – one for rapid signal transduction and another for slower adaptation (Shimizu, Tu and Berg 2010). The signal transduction module is composed of transmembrane chemoreceptors that change conformation upon ligand binding or other environmental perturbations and together with the adaptor protein CheW modulate the activity of a histidine kinase CheA (Parkinson, Hazelbauer and Falke 2015). Together with CheA and CheW, chemoreceptors form stable supramolecular sensory complexes that primarily cluster at cell poles in *E. coli* and other bacteria (Yang and Briegel 2020). The kinase CheA phosphorylates the diffusible response regulator CheY, which, when phosphorylated, binds to the flagellar motor to induce its clockwise rotation and thus cell tumbling. This signaling core is highly conserved among all chemotaxis pathways (Wuichet and Zhulin 2010). Many bacterial systems, including that of *E. coli*, also possess a specific phosphatase CheZ that rapidly dephosphorylates CheY, thereby ensuring that the phosphorylation level of CheY reflects the kinase activity with only short delay. In other chemotaxis pathways, CheY dephosphorylation is carried out by alternative phosphatases, CheC or CheX (Silversmith 2010). Some of the chemotaxis pathways, including the closely related pathway in *Salmonella enterica*, include an additional component of the sensory complexes CheV, which has the CheW-like scaffolding domain and the CheY-like regulatory domain (Alexander *et al*. 2010).

The signal transduction module of the chemotaxis pathway belongs to a larger family of two-component systems (TCSs) that enable environmental sensing in prokaryotes and are also present in fungi and plants (Stock, Robinson and Goudreau 2000). One important difference between the canonical TCSs and the chemotaxis pathways is that in the former the sensory, kinase and phosphatase activities are typically executed by a single sensory kinase protein, whereas in chemotaxis these functions are carried out by different proteins within one stable complex (Gumerov, Andrianova and Zhulin 2021; Sourjik and Armitage 2010). Such segregation of sensory and signaling activities likely facilitates evolutionary adaptation of the chemotaxis pathway to new environmental niches with different chemoeffector requirements, where specific chemoreceptors could be rapidly acquired or lost without affecting the function of the signaling core. Indeed, both specificities and the number of chemoreceptors apparently correlate with the respective lifestyles of bacterial species (Ortega, Zhulin and Krell 2017).

The chemotaxis pathway further includes an adaptation module that is composed of two enzymes CheR and CheB, which respectively methylate and demethylate specific residues on the receptor and thus counterbalance the effect of ligand binding on receptor conformation (Goy, Springer and Adler 1977; Kehry and Dahlquist 1982; Terwilliger, Wang and Koshland 1986). The (de)methylation rates depend primarily on the current activity of the receptor-kinase complex and they are slow compared to the other reactions within the pathway (Block, Segall and Berg 1983; Sourjik 2004; Sourjik and Berg 2002). Consequently, CheR and CheB provide a delayed integral negative feedback, which allows the cell to respond to temporal changes in experienced conditions over a wide range of backgrounds (Barkai and Leibler 1997; Berg and Purcell 1977; Kalinin *et al*. 2009; Lazova *et al*. 2011; Mesibov, Ordal and Adler 1973; Segall, Block and Berg 1986; Yi *et al*. 2000). This methylation-dependent adaptation module is unique in comparison to other TCSs, and present in the vast majority of bacterial chemotaxis pathways. In addition to CheR and CheB, adaptation in other chemotaxis pathways such as that of *Bacillus subtilis* involves CheV and a receptor deamidase CheD that are absent in *E. coli*, but the interplay between these different levels of adaptation remains poorly understood (Walukiewicz *et al*. 2014).

Clustering of chemoreceptors and associated chemotaxis proteins appears to be a universal feature of all studied prokaryotic chemotaxis systems (Sourjik 2004; Yang and Briegel 2020). Clustering allows receptor-kinase complexes to respond cooperatively and thus highly sensitively to changes in environmental conditions (Sourjik 2004; Tu 2013). Since receptors with different ligand specificities are mixed within clusters, clustering further facilitates signal integration (Parkinson, Ames and Studdert 2005). Finally, many bacteria express multiple chemotaxis systems, and hence spatial segregation provided by clustering might help to separate proteins belonging to different systems and thus prevent their undesired interference (Sourjik and Armitage 2010).

In contrast to this highly detailed molecular understanding of signal processing and motility control in *E. coli* and several other bacteria, the physiological importance of chemotaxis and flagellar motility are not well established even for model bacterial systems. In this review, we thus aim to summarize the current state of knowledge about different physiological aspects of chemotactic behavior. These range from importance of chemotaxis for enhanced nutrient acquisition by individual bacteria and population range expansion to the role that chemotaxis plays in bacteria-bacteria and bacteria-host interactions. We further illustrate how better understanding of bacterial chemotactic behavior in its physiological context(s) might help to rationalize many of its observed properties, from ligand specificity of chemoreceptors to growth-dependent regulation of motility gene expression.

## CHEMOTAXIS TOWARDS NUTRIENTS

### Correlation between chemotactic and nutritional preferences of bacteria

Early studies of chemotaxis showed that bacteria are attracted to common nutrients, such as amino acids or sugars (Adler, Hazelbauer and Dahl 1973; Mesibov and Adler 1972; Pfeffer 1884), while being repelled from harmful conditions such as toxic levels of inorganic ions or extreme pH (Tso and Adler 1974), which led to the assumption that bacteria use chemotaxis to accumulate in environmental niches that provide optimal conditions for growth. Confirming this correlation between chemotactic and metabolic preferences, several studies have shown that, even within the same chemical class, the most potent chemoattractants are those compounds that are also preferentially consumed (Neumann, Grosse and Sourjik 2012; Somavanshi, Ghosh and Sourjik 2016; Yang *et al*. 2015) or give the shortest lag time in growth when used as a carbon source (Fernandez *et al*. 2017). Thus, bacteria might generally utilize chemotaxis to enhance acquisition of high-value nutrients in their environment, and many ligand preferences of bacterial chemoreceptors could be explained by such nutrient taxis. Consistently, bacteria that acquired capabilities to metabolize environmental pollutants also apparently evolved chemotaxis towards these chemicals (Krell *et al*. 2013; Parales and Harwood 2002). More generally, evolutionary selection for locating optimal physiological conditions could explain responses to unconventional chemoeffectors, which signal by affecting metabolism or perturbing cellular physiology or energy state (Alexandre, Greer-Phillips and Zhulin 2004; Bi and Sourjik 2018; Schweinitzer and Josenhans 2010).

However, correlation between chemotactic and metabolic or physiological preferences of bacteria is not always the case. For instance, *B. subtilis* appears to use gradients of amino acids (Yang *et al*. 2015) or ethanol (Tohidifar *et al*. 2020) as environmental cues in order to locate sources of nutrients, such as plant roots or decaying organic matter, rather than because of their immediate nutritional value. As discussed below, such tactic responses to gradients of small molecules excreted by animals, plants or other microbes, irrespective of the nutritional value, might be common in host–bacteria interactions and enable bacteria to orient themselves relative to their hosts or cooperation partners and also to locate specific niches, such as wound areas.

### Adaptation of nutrient search strategies to respective environments

In the environment, bacteria are likely to encounter a variety of complex chemoeffector landscapes which result from different geometries of their release and diffusion, and from advection by flows and degradation (Raina *et al*. 2019). Typical examples are patches where an initial localized spike of attractant spreads by diffusion, e.g. after a burst of a phytoplankton cell. In turbulent marine environments, these patches can turn into filaments of nutrients (Taylor and Stocker 2012; Watteaux, Stocker and Taylor 2015). The simplest biologically relevant case is release of a freely diffusing attractant at a constant rate by a fixed point source – e.g. by a pore in a plant or animal epithelium or by a living cell aggregate – which results in a gradient where both concentration and the relative gradient of concentration (1/*c* d*c*/d*x*) decrease as the inverse of the distance to the source (Berg and Purcell 1977). In another relevant case, chemical concentration only varies in one direction, e.g. when an attractant is uniformly released from the flat surface of a large object or diffuses from an air-liquid interface. Uniform degradation of the attractant will result here in an exponential decay of attractant concentration away from the source, the steepness of which is determined by the degradation rate. In contrast, if the released chemical is absorbed by a distant sink, e.g. sessile (micro)organisms, a linear gradient of the chemical forms between source and sink. Importantly, these different gradient shapes will affect tactic behaviors. For instance, bacteria such as *E. coli* that respond to relative gradients (log-sensors) (Kalinin *et al*. 2009; Lazova *et al*. 2011; Menolascina *et al*. 2017; Mesibov, Ordal and Adler 1973; Sourjik and Berg 2002), will maintain constant velocity of chemotactic drift in an exponential gradient, while increasing drift velocity as they climb a point-source gradient or slowing down in a linear gradient. In the laboratory, most quantitative experiments probing chemotactic behaviors of swimming cells are carried out in steady linear gradients of chemoeffectors, which stems from the ease with which such gradients can be created within microfluidic devices (Ahmed, Shimizu and Stocker 2010; Colin, Zhang and Wilson 2014; Kalinin *et al*. 2009), as well as from the relative simplicity of the theoretical analysis of the cell behavior in that case. Nevertheless, more complex and time varying gradients can be mimicked under laboratory conditions by controlling flow in microfluidic devices (Ahmed, Shimizu and Stocker 2010; Englert, Manson and Jayaraman 2010; Stocker *et al*. 2008) or by releasing caged compounds to form transient patches (Brumley *et al*. 2019; Jikeli *et al*. 2015; Mccray and Trentham 1989).

**Figure 2.**
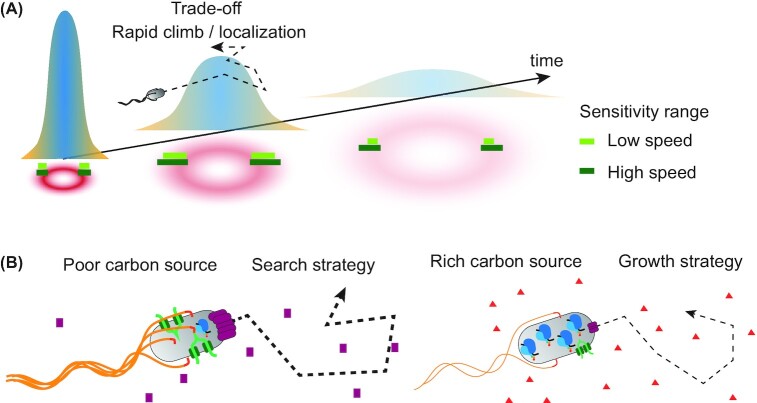
Trade-offs in chemotactic behavior and regulation of chemotaxis. **(A)**, Chemotactic response to time varying concentration profiles that could result from diffusive spreading of attractant patch needs to balance rapid gradient climbing and localization at the peak. Higher swimming velocity expands sensitivity range of bacterial chemotaxis, particularly in shallow gradients (right), but incurs additional energetic costs. **(B)**, Motility and nutrient uptake are regulated antagonistically with biosynthetic machinery dependent on the nutritional quality of the carbon source. During growth in poor carbon sources (left), motility is upregulated in proportion to potentially higher advantage provided by chemotaxis towards sources of additional nutrients (search strategy). In rich carbon sources (right), motility is downregulated to enable higher investment into biosynthetic machinery (growth strategy).

Responding to such dynamic gradients poses additional challenges to chemotactic bacteria (Fig. 2A), since they must not only climb the gradients rapidly but also localize near the maxima of attractant concentrations, and also react to the time evolution of the concentration profile, in order to maximize the efficiency of their chemotactic behavior (Blackburn and Fenchel 1999; Brumley *et al*. 2019; Stocker *et al*. 2008). Indeed, the pathway response of *E. coli* was suggested to meet a theoretical trade-off between efficient gradient climbing, ensured by the rapid tumble suppression upon stimulation, and localization at maximal concentrations, allowed by perfect adaptation (Clark and Grant 2005). This response was then shown to represent a generalist strategy to maximize the minimum nutrient uptake for any concentration profile, thus well-suited for unpredictable environments (Celani and Vergassola 2010). Consistent with this, the inferred distribution of gradient shapes that are most likely to be encountered by *E. coli* was found to be very wide (Clausznitzer *et al*. 2014). Another, complementary strategy to deal with spatiotemporally variable environments might be conferred by the large phenotypic variability of the chemotactic response observed even in clonal *E. coli* populations (Frankel *et al*. 2014; Karin and Alon 2021; Vladimirov *et al*. 2008).

Other chemotactic bacteria, e.g. those in marine habitats, might have further improved on this generalist strategy (Brumley *et al*. 2020). It was argued that marine bacteria have specifically adopted the run-reverse-flick motility pattern (Xie *et al*. 2011), which differs from *E. coli* run-tumble behavior, to improve their localization at nutrient maxima without compromising gradient climbing (Stocker *et al*. 2008; Xie and Wu 2014), although it might reduce their ability to explore new nutrient patches. A generally higher swimming speed and chemokinetic ability of marine bacteria might enable more efficient exploitation of transient nutrient patches, both increasing the localization at maxima (Son, Menolascina and Stocker 2016) and resulting in higher sensitivity to shallow gradients (Brumley *et al*. 2019; Hein *et al*. 2016). Finally, bacterial chemotaxis systems seem to be able to detect about as small a change in concentrations as possible, given physical limitations imposed by the cell size and by diffusion of ligands and of bacteria themselves (Aquino *et al*. 2011; Berg and Purcell 1977; Bialek and Setayeshgar 2005; Brumley *et al*. 2019; Colin, Zhang and Wilson 2014; Micali and Endres 2016; Mora and Wingreen 2010).

The physical properties of the environment can also affect cell swimming and chemotaxis beyond the aforementioned effects of flow on gradient shapes. Aquatic bacteria are particularly exposed to fluid flows that exert mechanical shear, which can stir the swimming direction (Jing *et al*. 2020; Marcos *et al*. 2012) and drive swimming bacteria to regions of high flow shear (Bearon and Hazel 2015; Rusconi, Guasto and Stocker 2014). Because of this stirring, shear flows were predicted (Bearon and Pedley 2000; Locsei and Pedley 2009; Luchsinger, Bergersen and Mitchell 1999) and observed (Rusconi, Guasto and Stocker 2014) to reduce the efficiency of chemotaxis, even when the gradient is unaffected. Here as well, the run-reverse swimming pattern might bring the adaptive advantage of improving the chemotactic response in flow (Luchsinger, Bergersen and Mitchell 1999). Bacteria can also exploit physical properties of the environment to improve chemotactic navigation. A prominent example is provided by magnetotactic bacteria, which use needle-shaped magnetosomes to align with the earth magnetic field (Blakemore 1982; Faivre and Schuler 2008) and to follow it downwards (Blakemore, Frankel and Kalmijn 1980; Simmons, Bazylinski and Edwards 2006; Zhang *et al*. 2010). Combined with aerotaxis, such magnetotaxis enables these bacteria to position themselves in the growth-favorable microaerobic layer of their aqueous sediment habitats (Faivre and Schuler 2008; Lefevre and Bazylinski 2013; Mao *et al*. 2014; Spormann and Wolfe 1984; Yazi *et al*. 2018; Zhang *et al*. 2010).

### Trade-offs between motility and growth

Although accumulation towards sources of nutrients may lead to increased nutrient uptake by bacteria and therefore to enhanced growth, swimming motility also requires high investment of cellular resources. Biogenesis of motility system and powering of flagellar motor rotation consume respectively up to several percent of total cellular protein and energy budget in *E. coli* (Berg 2003; Colin and Sourjik 2017; Milo *et al*. 2010) and these costs are likely similar or even higher in other bacteria. Consequently, expression of motility genes can significantly reduce bacterial growth under conditions where it provides no advantage (e.g. in a well-stirred environment), implying the existence of an environment-dependent fitness trade-off between the benefits and costs of resource investment in motility (Fraebel *et al*. 2017; Ni *et al*. 2020; Ni *et al*. 2017; Taylor and Stocker 2012; Yi and Dean 2016). Such adaptive trade-offs are common in bacteria, as well as in other organisms, which typically need to optimize different conflicting functions during evolution (Ferenci 2016). Indeed, besides trade-offs associated with gene expression, other trade-offs related to the costs of precise operation of the chemotaxis machinery have been recognized (Brumley *et al*. 2019; Govern and Ten Wolde 2014; Lan *et al*. 2012).

As a consequence, bacteria evolved multiple regulatory strategies to optimize cellular resource allocation dependent on their growth conditions (Molenaar *et al*. 2009; Schuetz *et al*. 2012; Scott *et al*. 2010).This is reflected in the multilevel regulation of expression of bacterial flagellar and chemotaxis genes by a variety of environmental and cellular cues (Amsler, Cho and Matsumura 1993; Chevance and Hughes 2008; Guttenplan and Kearns 2013; Pruss 2017). One of the most prominent mechanisms of this regulation in *E. coli* is by the carbon catabolite repression mediated by cyclic adenosine monophosphate (cAMP), which reflects growth rate and carbon uptake into the cell and is elevated during growth on poor carbon sources (Adler and Templeton 1967; Hui *et al*. 2015; Liu *et al*. 2005). High levels of cAMP under carbon-limited growth activate multiple pathways for uptake and catabolism of alternative carbon sources, as well as genes involved in the TCA cycle and amino acid synthesis (Hui *et al*. 2015; Liu *et al*. 2005). This regulation enhances nutrient uptake and catabolism at a cost of reduced allocation of resources in protein biosynthesis (You *et al*. 2013). The activation of flagellar and chemotaxis genes by cAMP might follow similar regulatory logic, enhancing carbon acquisition by active accumulation towards sources of nutrients in carbon-poor environments (Amsler, Cho and Matsumura 1993; Hui *et al*. 2015; Liu *et al*. 2005) (Fig. 2B). Indeed, active acquisition of nutrients by motile bacteria becomes increasingly important in carbon-poor environments, as demonstrated by co-culturing chemotactic and non-chemotactic *E. coli* in presence of nutrient gradients (Ni *et al*. 2020). Notably, the relative fitness benefit provided by chemotaxis exhibits the same dependence on the growth rate as expression of flagellar genes, indicating that *E. coli* invests resources in motile behavior in proportion to its anticipated benefit. A fitness benefit of chemotaxis in an unstirred co-culture was also observed in absence of artificially introduced gradients of nutrients, apparently due to the self-generation of gradients by bacteria through excretion and subsequent chemotaxis-mediated consumption of metabolites (Ni *et al*. 2020). Similar cross-feeding might contribute to positive selection for motility in natural microbial communities, and it might also explain the rapid accumulation of motility-activating mutations in a resting culture of *E. coli* (Parker, Demetci and Li 2019).

In bacteria, trade-offs associated with resource allocation are typically adaptive and can be tuned by mutations dependent on the environment (Ferenci 2016). Consistently, under experimental selection for enhanced chemotaxis, the balance between bacterial motility and growth could be easily and gradually shifted by a variety of mutations along a well-defined growth-motility trade-off line (Fraebel *et al*. 2017; Ni *et al*. 2017; Yi and Dean 2016). The main phenotypic change observed in these different studies was an enhancement of flagellar gene expression and thus of cell swimming velocity, likely because of the steep dependence of the chemotactic drift of individual bacteria on their velocity (Schauer *et al*. 2018). Moreover, a similar trade-off was observed between enhancement of motility and growth reduction in all studies. In contrast, genetic mutations underlying the evolved phenotypic changes differed largely between individual studies, presumably due to the epistatic effects of strain background and/or differences in selection protocols. This confirms the high plasticity of bacterial motility that enables it to evolutionarily adapt to novel environments, which might be a common property of bacterial networks (Hindre *et al*. 2012). Notably, it was proposed that such evolvability might be favored by the hierarchical design of bacterial flagellar gene regulatory networks (Ni *et al*. 2017).

## IMPORTANCE OF VARIABILITY OF CHEMOTACTIC PERFORMANCE IN BACTERIAL POPULATIONS

Trade-offs between different conflicting functions might also explain the co-existence of individual cells with different physiological states within bacterial populations. The importance of such phenotypic heterogeneity that is observed for behaviors of individual cells even in genetically homogeneous microbial populations has been well recognized in recent years (Bettenworth *et al*. 2019; Jung *et al*. 2019; Veening, Smits and Kuipers 2008; Weigel and Dersch 2018). Bacterial swimming behavior provided one of the first examples of such behavioral individuality (Spudich and Koshland 1976). In *E. coli*, both the run-and-tumble bias and the chemotactic sensitivity are subject to cell-to-cell variability as well as temporal variability within each cell. Variations in the expression level of chemotactic proteins, particularly CheR and CheB (Dufour *et al*. 2016) appear to produce a cell-to-cell variability in the adaptation dynamics (Keegstra *et al*. 2017) and in the duration of runs (Min *et al*. 2009). Flagellar number, which varies among individual cells with the expression levels of motility genes, is another modulator of the tumbling rate (Mears *et al*. 2014; Vladimirov, Lebiedz and Sourjik 2010). The pathway gain, arising from cooperative responses of the chemoreceptor clusters and flagellar motors, also appears to show strong cell-to-cell variability (Salek *et al*. 2019).

This variability in the settings of the chemotaxis pathway strongly modulates the chemotactic performance of individual cells (Salek *et al*. 2019; Waite *et al*. 2016; Wong-Ng *et al*. 2016). This is thought to allow bet-hedging strategies in response to chemical gradients, with the population separating between adventurous strong responders and more sedentary weak responders. Such separation of the population has indeed been observed in self-generated chemical gradients (Fu *et al*. 2018; Salek *et al*. 2019). Since the set of pathway parameters eliciting the strongest response varies strongly with gradient shape and steepness (Dufour *et al*. 2014; Long, Zucker and Emonet 2017), it was argued that phenotypic heterogeneity in chemotactic behavior might have been evolutionarily selected to optimize chemotaxis in variable environments (Frankel *et al*. 2014; Karin and Alon 2021; Vladimirov *et al*. 2008). The variability in pathway activity was, however, found to be reduced at high levels of chemoattractant, allowing cell populations to combine the exploratory strategy in nutrient-poor media with the faithful and fairly homogeneous response once gradients are encountered (Kamino *et al*.^2020^).

Additionally, even within unstimulated single cells, slow but large temporal fluctuations of CheY phosphorylation and therefore of the tumbling rate could be observed (Colin *et al*. 2017; Keegstra *et al*. 2017; Min *et al*. 2012; Min *et al*. 2009). These temporal fluctuations apparently originate from the amplification of the noisy and slow receptor methylation dynamics as well as of thermal noise by strongly coupled receptor clusters (Colin *et al*. 2017), and they can largely explain the broad, power-law distributions of run durations observed in cell populations (Korobkova *et al*. 2004; Min *et al*. 2009; Park *et al*. 2010). Such long-term fluctuations are thought to enhance the effective diffusion of single cells and therefore their ability to explore the environment (Benichou *et al*. 2011; Matthaus, Jagodic and Dobnikar 2009; Matthaus *et al*. 2011), and they might also enhance the chemotactic drift (Flores *et al*. 2012). Additionally, the activity fluctuations were predicted to increase the coordination of the flagellar motors, thus improving chemotactic performance (Sneddon, Pontius and Emonet 2012).

Even more pronounced heterogeneity is observed in other cases, where only a fraction of cells in a population becomes motile. Such bimodality of flagellar gene expression has been described in *B. subtilis* (Kearns and Losick 2005), *S. enterica* serovar Typhimurium (Koirala *et al*. 2014) as well as in pathogenic *E. coli* strains (Laganenka *et al*. 2020), and it is likely to be a common phenomenon. Differentiation of the population into distinct subpopulations of motile and sessile non-flagellated cells might reflect previously discussed trade-offs/physiological conflicts between colonization and exploration of the environment (Koirala *et al*. 2014; Mukherjee and Kearns 2014) and between resource investment in growth and motility (Syvertsson *et al*. 2021). Yet another trade-off exists in bacterial pathogens, where flagellar motility and chemotaxis provide benefit at the early stage of infection (see below) but flagellation later becomes a burden since the flagellum is a major antigen recognized by the immune system (Sporing *et al*. 2018). Consistently, relative fractions of motile cells in bacterial populations are regulated by a variety of factors, from nutrient levels and stress response to physical properties of the environment, but the complex underlying mechanisms remain only partly understood (Koirala *et al*. 2014; Laganenka *et al*. 2020; Mukherjee and Kearns 2014; Sporing *et al*. 2018; Wang *et al*. 2020).

## MOTILITY AND CHEMOTAXIS IN POPULATION AND COLLECTIVE BEHAVIORS

### Motility-driven expansion of the population range

**Figure 3.**
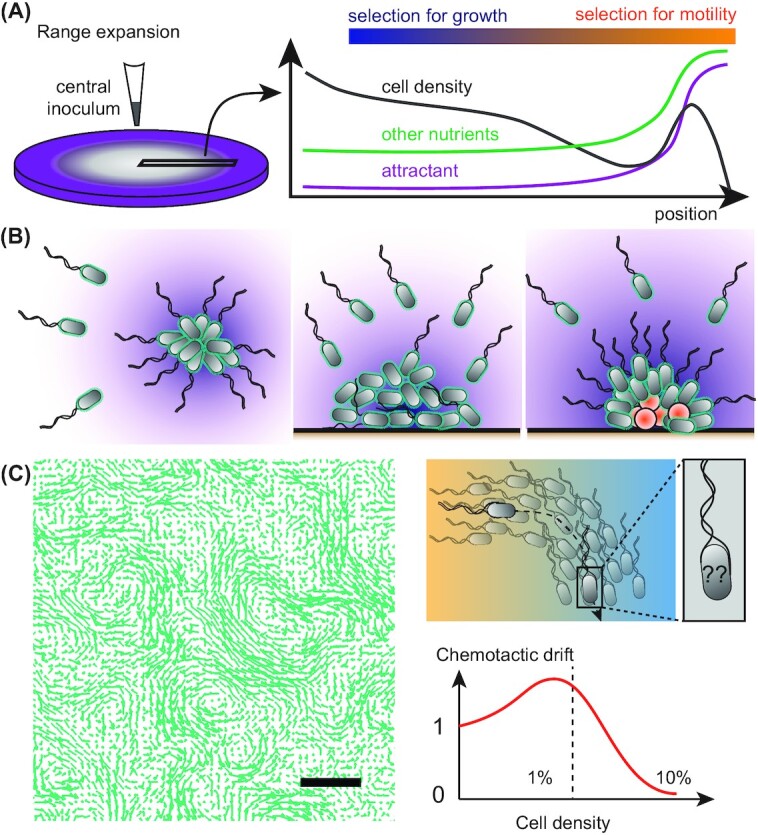
Collective chemotactic behaviors. **(A)**, Population expansion driven by chemotaxis towards self-generated gradients produced by metabolite consumption in porous medium (left) results in a spatial organization of the cell population, with selection for motility at the front and for growth at the rear of the spreading colony (right). **(B)**, Chemoattraction to quorum-sensing signals can enhance autoaggregation and biofilm formation in single or multi-species communities of bacteria that secrete an attractant. **(C)**, Swirling collective motion emerges at high bacterial cell densities, as observed on maps of the local cell velocities (left). It impairs the chemotactic perception of gradients by inducing random reorientations on the time scale of gradient sensing (top right), thus reducing chemotactic drift above the cell density at which collective motion begin to emerge (bottom right, dashed line).

Colonization of a porous growth medium from a single inoculation site represents the simplest example of chemotactic behaviour at the level of a bacterial population. Such motility-dependent expansion of the bacterial population range is typically experimentally studied using a soft agar assay, where bacteria grow and propagate from a central inoculum into a low percentage agar gel supplied with nutrients (Adler 1966) (Fig. 3A). The agar mesh is loose enough for the cells to swim and navigate gradients, such that colony propagation results from a combination of growth, motility and chemotaxis. In contrast to liquid media, swimmers get stuck in the agar mesh, which they can only escape by tumbling, therefore restricting efficient cell propagation to cells with intermediate tumbling rates (Wolfe and Berg 1989). Although chemotaxis is not strictly required for spreading in soft agar, it can greatly accelerate the rate of bacterial colony expansion. By consuming nutrients and other chemicals inside the colony, metabolically active bacteria create gradients in the medium, which they can subsequently use to migrate outwards as expanding chemotactic rings of a constant high density (Adler 1966; Koster *et al*. 2012). This behavior can be mathematically captured by the classical Keller–Segel model of chemotaxis and its numerous variants (Keller and Segel 1971; Tindall *et al*. 2008). Similar behavior can also be observed in liquid media, for example in narrow straight channels (e.g. glass capillaries) where the population response to a self-generated gradient takes the form of travelling chemotactic bands (Adler 1966; Saragosti *et al*. 2011).

Although such traveling bands formed by bacterial monoculture are unlikely to be common in nature, they might nevertheless be relevant for population spreading in natural porous environments. Moreover, these assays have been used as a general model to investigate the interplay between growth and directed movement in collective range expansion of a population. In this model, the chemoattractants can serve as aroma-like cues that allow a population to migrate outward well before nutrients run out, even if these attractants make up only a small portion of the nutrients available in the medium (Cremer *et al*. 2019). Moreover, collectively spreading bacterial populations are able to cross and colonize maze-like or even fractal structures that mimic environments with complex geometries such as the soil or the lungs (Park *et al*. 2003; Phan *et al*. 2020). The importance of heterogeneity in individual chemotactic responses within spreading populations was also investigated. It was shown that the apparent conflict between this cell-to-cell variability and the coherent motion as a band of the population can be resolved by the stratification of the individuals in the self-generated chemical gradient according to their individual characteristics (Fu *et al*. 2018).

Motility and chemotaxis in a porous medium further play an important role when several populations compete for the same habitat. Trade-off between growth and expansion rate can in this case lead to phenotypic segregation, where fast swimming but slow growing cells occupy the outer rings, while fast growing but slow swimming cells keep the center and are at risk of being globally outcompeted over the whole plate (Gude *et al*. 2020; Liu *et al*. 2019) (Fig. 3A). Consistently, in an initially clonal population there is selection for mutations that favor swimming speed at the expense of growth in the outer edge of the spreading colony, whereas mutations favoring growth over speed are selected close to the center (Liu *et al*. 2019; Ni *et al*. 2017). Thus, the observed trade-offs between growth and motility likely play an important role in bacterial niche formation and evolution in structured environments. Strikingly, in *E. coli* these trade-offs lead to a negative frequency selection on strains with different levels of motility in a spreading population, resulting in stable coexistence of such strains (Gude *et al*. 2020). Spatiotemporal structuring of multispecies bacterial colonies can be further greatly enhanced by either antagonistic or cooperative regulatory interactions between species (Curatolo *et al*. 2020).

### Autoaggregation of chemotactic bacteria

Besides these dynamic traveling bands that rely on nutrient gradient formation and reshaping by a spreading bacterial population, chemotactic bands can also emerge through several other mechanisms. At the levels of individual bacteria, accumulation towards a specific location can be observed when cells respond to opposite gradients of chemicals, as well as in response to oxygen (Alexandre, Greer and Zhulin 2000; Rebbapragada *et al*. 1997; Shioi *et al*. 1988), pH (Yang and Sourjik 2012) or temperature (Oleksiuk *et al*. 2011; Paulick *et al*. 2017; Salman and Libchaber 2007; Yoney and Salman 2015), for which the chemotactic response changes from repellent to attractant as a function of the level of stimulation. In this scenario, two opposing chemotactic ‘forces’ drive bacteria to accumulate in an intermediate region, where the net chemotactic velocity is null (Hu and Tu 2013; Zhang *et al*. 2019). Bidirectional sensing of many physical and chemical stimuli (e.g. pH or temperature) by chemotactic bacteria is thought to generally play an important role in locating physiologically optimal niches in chemically complex environments.

Emergence of higher complexity patterns, which can occur even in initially uniform environments, typically relies on tactic responses elicited by chemical interactions between bacteria, although other mechanisms such as swimming speed regulation might contribute, too (Cates 2012; Curatolo *et al*. 2020). Instead of (or in addition to) generating and sensing gradients of chemicals which were already present in the medium, bacteria release chemicals that elicit chemotactic responses by their peers, which can lead to chemotactic self-attraction and generate aggregative processes on various length scales. For example, *E. coli* forms regular lattices of millimeter-large high-density spots on soft agar plates, which could be explained by the chemotactic response to self-produced gradient of aspartate (Budrene and Berg 1991; Budrene and Berg 1995). Chemotaxis to self-produced attractants can further lead to accumulation of bacteria in small sub-millimeter cavities in microfluidic devices (Park *et al*. 2003).

Although in the aforementioned examples cluster formation does not require any physical interactions among bacteria, self-attraction may also enhance bacterial aggregation (clumping) that is mediated by various surface adhesins (Defoirdt 2011) (Fig. 3B). Indeed, chemotactic response to the quorum-sensing molecule autoinducer 2 (AI-2) secreted by cell aggregates can indeed largely enhance the autoaggregation of *E. coli*, mediated either by adhesin antigen 43 or by curli filaments (Hegde *et al*. 2011; Jani *et al*. 2017; Laganenka, Colin and Sourjik 2016; Song and Wood 2021). AI-2 is an interspecies communication signal produced by a wide variety of bacteria (Pereira, Thompson and Xavier 2013; Waters and Bassler 2005). Further supporting its potential role in establishing interspecies interactions within complex microbial communities, chemoattraction to AI-2 can mediate co-aggregation of different species (Laganenka and Sourjik 2017), and it is not restricted to AI-2-producing bacteria (Zhang *et al*. 2020). Besides AI-2, other quorum-sensing signals can also mediate bacterial self-attraction responses, for instance the S signal in *Vibrio parahaemolyticus* (Lamb, Trimble and McCarter 2019). Chemotaxis can also mediate self-repulsion. For example, the chemotaxis pathway of *Azospirillum brasilense* exerts a negative effect on cell clumping (Bible *et al*. 2008). The complex chemotactic response of *E. coli* to self-produced indole might serve both functions, mediating self-repulsion at low levels of secreted indole but leading to self-attraction when the levels are high (Yang *et al*. 2020). Given the large diversity of autoinducer signals produced by bacteria, all these different scenarios of intra- and interspecies attraction and repulsion are likely to be found in natural microbial communities where they might produce complex collective behaviors (Grauer *et al*. 2020).

### Physical interactions between motile cells

In addition to chemical and adhesive interactions, at higher densities cell swimming itself leads to physical interactions between bacteria, both direct when cells collide (steric interactions) and through the fluid that they displace (hydrodynamic interactions). When the cell density increases, hydrodynamic interactions are the main contributor to the emergence of swirling collective motion made of intermittent jets and eddies of hundreds of cells (Cisneros *et al*. 2007; Dunkel *et al*. 2013; Koch and Subramanian 2011; Liu *et al*. 2000; Luchsinger, Bergersen and Mitchell 1999; Sokolov *et al*. 2007; Wensink *et al*. 2012; Wolgemuth 2008). These are observed in many bacterial species at the front of swarms of swimming bacteria propagating at the surface of semi-solid agar gels (Be'er and Ariel 2019; Kearns 2010; Partridge and Harshey 2013) or near air–water interfaces (Holscher *et al*. 2015). Interestingly, these physical interactions strongly modify the chemotactic response, both in two- and three-dimensional geometries: Although the response is slightly enhanced at moderate cell densities, the emergence of the collective motion annihilates the ability of *E. coli* to follow chemical gradients (Colin, Drescher and Sourjik 2019), due to rapid randomization of the direction of motion of swimming bacteria caused by collective motion that prevents gradient sensing through temporal comparisons (Fig. 3C). Therefore, there is a physical upper limit on the density at which bacteria can chemotactically accumulate near a source of attractant or in a travelling chemotactic band. Such reduction of chemosensing at high density must also affect bacterial swarms, where collective motion arises in the dense quasi-monolayer of swimming cells behind the colony edge (Darnton *et al*. 2010; Harshey 1994; Jeckel *et al*. 2019; Kearns and Losick 2003). Accordingly, chemotaxis is not necessary for swarming (Ariel *et al*. 2018; Be'er and Ariel 2019; Burkart, Toguchi and Harshey 1998; Sidortsov, Morgenstern and Be'er 2017). Nevertheless, a recent report suggested that an *E. coli* swarm may be able to bias its motion towards higher concentrations of an attractant (Tian *et al*. 2021), which would require specific mechanisms to counteract the physics-driven loss of chemotaxis. These could include a strongly reduced tumbling rate, which is observed not only for *E. coli* but also for other swarming bacteria (Ford *et al*. 2018; Mariconda, Wang and Harshey 2006; Partridge *et al*. 2019; Partridge *et al*. 2020), as well as cell elongation (Ilkanaiv *et al*. 2017; Kearns 2010) and modified fluid flows within the swarm compared to suspensions (Chen *et al*. 2017; Jeckel *et al*. 2019; Li *et al*. 2017). Importantly, these results hold for the flagella-propelled bacteria. In contrast, the chemotactic ability of bacteria that move at high density on semisolid surfaces using twitching or gliding motility remains poorly understood, although their collective migration up chemical gradients has been reported (Guzzo *et al*. 2018; Islam and Mignot 2015; Kearns, Robinson and Shimkets 2001; Oliveira, Fostera and Durham 2016; Sampedro *et al*. 2015).

### Roles of flagella, motility and chemotaxis in biofilm formation

With the formation of a surface attached biofilm, bacteria adopt a sessile lifestyle which offers protection against harsh environments and enables division of labor in bacterial communities. The formation of submerged biofilms on liquid–solid interfaces typically proceeds through several stages, including surface attachment, growth and maturation of matrix-embedded communities, and finally biofilm dispersion (Rumbaugh and Sauer 2020; Stoodley *et al*. 2002). These sessile biofilm communities are commonly viewed in opposition to the explorative motile planktonic lifestyle. Indeed, genes required for production of biofilm matrix and those for motility are antagonistically regulated in *E. coli* and other bacteria, including their mutually exclusive expression (Besharova *et al*. 2016; Guttenplan and Kearns 2013; Pesavento *et al*. 2008; Pruss 2017; Serra *et al*. 2013). A major signal controlling this transition between gene expression profiles characteristic for motile and sessile states is the second messenger cyclic diguanosine monophosphate (c-di-GMP), which generally promotes biofilm formation and reduces motility (Guttenplan and Kearns 2013; Hengge 2009; Jenal and Malone 2006). Besides leading to repression of flagellar gene expression, c-di-GMP can also reduce motility at the post-translational level (Guttenplan and Kearns 2013), e.g. by activating the flagellar motor break protein YcgR in *E. coli* (Boehm *et al*. 2010; Paul *et al*. 2010; Ryjenkov *et al*. 2006).

Despite this generally antagonistic regulation, increasing evidence suggests that flagella, motility and chemotaxis play important roles at all stages of biofilm formation. Strains of *E. coli* defective in motility form smaller and sparser submerged biofilms (Pratt and Kolter 1998; Wood *et al*. 2006). Similar phenotypes were observed for non-motile mutants of diverse bacteria, including *Pseudomonas*, *Shewanella*, *Agrobacterium* and *Bacillus* species (Holscher *et al*. 2015; Merritt, Danhorn and Fuqua 2007; O'Toole and Kolter 1998; Thormann *et al*. 2004). Flagellar motility indeed strongly promotes the initial attachment to the surface (Berke *et al*. 2008; Elgeti and Gompper 2013; Li *et al*. 2011). Attachment might be further enhanced by a strong chemoattractant response that suppresses tumbles, thus physically favoring accumulation of swimming bacteria at the surface and increasing their chances of attachment (Berke *et al*. 2008; Li and Tang 2009; Suchanek *et al*. 2020). Motility and chemotaxis can further drive bacteria towards a favorable niche for attachment and/or biofilm formation if the latter is chemoattractive, e.g. an air–water interface (Ardre *et al*. 2015; Holscher *et al*. 2015), gut epithelial surface (Misselwitz *et al*. 2012) or plant root (Scharf, Hynes and Alexandre 2016). Besides enhancing accumulation and attachment to surfaces, chemotaxis to the self-produced attractant AI-2 (see above) promotes the formation of larger and more structured submerged *E. coli* biofilms (Jani *et al*. 2017; Laganenka, Colin and Sourjik 2016). Since AI-2 is produced by many bacteria and mediates cross-species chemical interactions (Pereira, Thompson and Xavier 2013), chemoattraction to AI-2 may also favor co-aggregation and mixed biofilm formation, as indeed observed in co-cultures of *E. coli* and *E. faecalis* (Laganenka and Sourjik 2017). In addition to the requirement of motility for such chemotaxis-dependent enhancement of biofilm formation, flagella can directly promote surface attachment, serving as adhesins (Friedlander, Vogel and Aizenberg 2015).

In the mature biofilm, motility is repressed but the flagellum could be repurposed as an important structural element of the biofilm matrix (Besharova *et al*. 2016; Serra *et al*. 2013; Wood *et al*. 2006). Finally, reactivation of motility may be important at the stage of biofilm dispersal (Rumbaugh and Sauer 2020), which might be further enhanced by chemotactic self-repulsion. Interestingly, in *Helicobacter pylori* this self-repulsion is mediated by AI-2 (Anderson *et al*. 2015; Rader *et al*. 2011; Sweeney *et al*. 2019), in contrast to its biofilm-promoting role as an attractant in *E. coli*.

## MOTILITY AND CHEMOTAXIS IN HOST-MICROBE INTERACTIONS

### Colonization and infection of animal hosts

**Figure 4.**
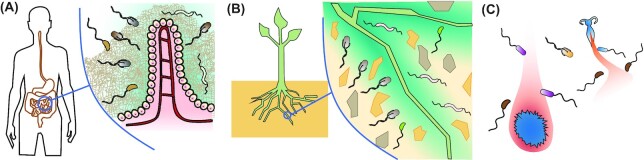
Relevance of chemotactic motility in natural bacterial habitats. **(A)**, In the gut, bacteria navigate in the lumen according to chemical gradients emanating from the epithelium. Motility is further used to penetrate the mucus layer that surrounds the epithelium. **(B)**, In the rhizosphere, bacteria navigate through the complex structure of the soil and follow chemical gradients released by plant roots. **(C)**, In the marine environment, bacteria follow chemical gradients released by planktonic and larger organisms and face turbulent flows which stir both the gradients and the cells as they swim.

Although only a fraction of bacteria associated with animal hosts are motile, flagellar motility and chemotaxis are common among bacterial pathogens, and typically important for successful host colonization and infection (Chaban, Hughes and Beeby 2015; Erhardt 2016; Matilla and Krell 2018). Motility might have several functions in the animal-microbe interactions (Chaban, Hughes and Beeby 2015; Erhardt 2016), and it might be particularly important in the gastrointestinal (GI) tract, where most of the animal microbiota resides (Fan and Pedersen 2021) (Fig. 4A). Successful colonization of the GI tract by enteric bacteria mostly depends on their ability to penetrate (or disrupt) the viscous mucus layer to reach a favorable niche. The importance of the mucus barrier in maintaining gut homeostasis is underscored by studies showing that MUC2-deficient mice are prone to spontaneous inflammation (Van der Sluis *et al*. 2006) and less resistant to bacterial infection (Bergstrom *et al*. 2010; Zarepour *et al*. 2013). Flagellar motility enables bacteria to increase their rate of encounter with the mucus surface (Misselwitz *et al*. 2012) and attach to and penetrate the mucous layer (Arora *et al*. 1998; Baban *et al*. 2013; Lane *et al*. 2005; Pichon *et al*. 2009; Tamar, Koler and Vaknin 2016; Wright, Seed and Hultgren 2005). Whereas some bacteria degrade or modify the mucus to facilitate penetration (Celli *et al*. 2009; Szabady *et al*. 2011), others (like *S*. Typhimurium) preferentially invade epithelial cells in the gut regions devoid of a continuous mucus layer (Furter *et al*. 2019). The attachment rate can be further enhanced by epithelium-produced molecules like mucins and their degradation products (Hugdahl, Beery and Doyle 1988; Nelson *et al*. 1990) or, potentially, AI-2 mimics (Ismail, Valastyan and Bassler 2016), which is consistent with a growing appreciation for the role of compounds released into the lumen by endocrine and immune systems of animal hosts in modulating host-microbe interactions during gut colonization (Neuman *et al*. 2015; Pacheco and Sperandio 2009; Rhee, Pothoulakis and Mayer 2009). It is likely that some of these molecules also mediate chemotactic responses. Specifically, *E. coli* has been shown to sense a number of human hormones, including norepinephrine (NE), 3,4-dihydroxymandelic acid (DHMA), dopamine, melatonin, as well as other chemicals that might be secreted into the gut lumen by animal hosts and/or microbiota, such as spermidine and indole (Bansal *et al*. 2007; Lopes and Sourjik 2018; Pasupuleti *et al*. 2014; Pasupuleti *et al*. 2018; Sule *et al*. 2017). Interestingly, responses to several of these compounds mediated by two major *E. coli* chemoreceptors, Tar and Tsr, are opposite (Lopes and Sourjik 2018; Yang *et al*. 2020). As discussed above, such opposing responses could lead to bacterial accumulation at an intermediate point within a gradient, which could be at a certain distance from gut epithelial surface, possibly enabling *E. coli* bacteria to escape antimicrobial activities of the mucous layer while remaining in proximity to the epithelium (Lopes and Sourjik 2018). Alternatively, a bimodal response could result in avoidance of intermediate concentrations, as appears to be the case for indole response of *E. coli* and was proposed to split bacteria into two subpopulations, one attracted towards the source of chemoeffector and the other repelled from it (Yang *et al*. 2020). Indeed, chemotaxis towards hormones or repulsion from indole was proposed to enhance attachment of *E. coli* to HeLa cells (Bansal *et al*. 2007; Bansal *et al*. 2008).

The involvement of motility and chemotaxis in the establishment and maintenance of infection is well documented for a number of human pathogens, see (Matilla and Krell 2018) for a recent comprehensive review. The best studied examples of chemotaxis in motile pathogenic bacteria include *Helicobacter pylori* (Aihara *et al*. 2014; Collins *et al*. 2018; Hanyu *et al*. 2019; Huang *et al*. 2015; Johnson and Ottemann 2018; Perkins *et al*. 2019; Schweinitzer *et al*. 2008; Williams *et al*. 2007), *Campylobacter jejuni* (Elgamoudi *et al*. 2021; Khan *et al*. 2020; Korolik 2019; Li *et al*. 2014), *S*. Typhimurium (Olsen *et al*. 2013; Rivera-Chavez *et al*. 2016; Stecher *et al*. 2008; Stecher *et al*. 2004), *Vibrio cholerae* (Echazarreta and Klose 2019; Freter, O'Brien and Macsai 1981), and *Pseudomonas aeruginosa* (Corral-Lugo *et al*. 2018; Garvis *et al*. 2009; Martin-Mora *et al*. 2018; Matilla *et al*. 2021; Reyes-Darias *et al*. 2015; Rico-Jimenez *et al*. 2016; Schwarzer, Fischer and Machen 2016). All of these pathogens exhibit chemotactic responses to at least some of the common metabolites, such as amino acids, sugars and organic acids. This indicates that, similar to *E. coli*, their chemoeffector preferences could be at least partly explained by the role of chemotaxis in enhanced nutrient acquisition, although some of these metabolites might play double roles as nutrients and as cues released by the host. Among these pathogenic bacteria, the most specialized spectrum of chemoeffector metabolites is observed for *H. pylori* that infects gastric epithelium and is therefore adapted to a highly specific ecological niche (Johnson and Ottemann 2018), whereas the broadest spectrum of metabolites is recognized by the opportunistic and highly versatile pathogen *P. aeruginosa* (Matilla and Krell 2018; Ortega, Zhulin and Krell 2017). Nevertheless, many tactic responses of pathogens appear to primarily or exclusively serve as orientation cues within their animal hosts, enabling bacteria to locate sites of infection. These include taxis to urea, pH and bicarbonate by *H. pylori* (Cerda, Rivas and Toledo 2003; Huang *et al*. 2017; Huang *et al*. 2015), to deoxycholate by *C. jejuni* (Li *et al*. 2014), and to histamine, gamma-aminobutyrate (GABA) and inorganic phosphate by *P. aeruginosa* (Corral-Lugo *et al*. 2018; Reyes-Darias *et al*. 2015; Rico-Jimenez *et al*. 2016), as well as aero- and energy taxes exhibited by most pathogens (Behrens *et al*. 2013; Horne, Mattson and Pruss 2009; Rivera-Chavez *et al*. 2013; Vegge *et al*. 2009). It is worth noting that the ability of at least some bacterial pathogens to benefit from chemotaxis during infection depends on the environmental context. The role of chemotaxis in *S*. Typhimurium infection only becomes apparent with the advent of intestinal inflammation (Stecher *et al*. 2008) and *V. cholerae* strains lacking particular chemotaxis gene clusters show higher fitness compared to the wild type cells in the proximal small intestine (Butler and Camilli 2004; Millet *et al*. 2014). Given that much of the complexity of metabolic interactions and chemical communication between animal hosts and their microbiota remains to be uncovered, the aforementioned host-specific tactic responses likely represent only a fraction of cues and signals perceived by pathogenic as well as by non-pathogenic motile bacteria inhabiting animal guts.

### Interactions in the rhizosphere and in aquatic environments

With its porous structure and variable water and nutrient content, soil represents a much more heterogeneous and complex environment. Since it typically sustains less fluid flow and has a lower density of microorganisms compared to the animal GI tract, soil can support stable long-range gradients of nutrients and signaling molecules. Together with the fact that soil is typically also more nutrient-poor, it is therefore not surprising that swimming motility and chemotaxis are common among soil bacteria (Fig. 4B). Motility seems to play a particularly important role in the rhizosphere, the region of the soil immediately surrounding plant roots that is enriched in root exudates and active microbial communities. Accordingly, plant pathogens and symbionts are nearly all motile and possess on average nearly twice as many chemoreceptors as motile animal/human pathogens (Lacal *et al*. 2010; Matilla and Krell 2018; Scharf, Hynes and Alexandre 2016). Chemotaxis in the rhizosphere can indeed enhance both symbiotic and pathogenic interactions of bacteria with their plant hosts through chemotaxis towards a variety of root- or leaf-secreted chemicals, including organic acids, carbohydrates, sugar alcohols, amino acids and plant hormones, for recent reviews see (Matilla and Krell 2018; Scharf, Hynes and Alexandre 2016). Flavonoids, phenolic compounds secreted by plant roots, have also been proposed to serve as specific chemoattractants (Dharmatilake and Bauer 1992), although these findings were recently questioned (Compton *et al*. 2020).

The importance of chemotaxis towards root exudates and specific compounds for colonization is well documented in several host–symbiont systems, most prominently *Sinorhizobium meliloti* (Caetano-Anolles, Crist-Estes and Bauer 1988; Caetano-Anolles, Wrobel-Boerner and Bauer 1992; Gulash *et al*. 1984), *Rhizobium leguminosarum* (Miller *et al*. 2007; Yost, Rochepeau and Hynes 1998) and *A. brasilense* (Greer-Phillips, Stephens and Alexandre 2004; O'Neal, Vo and Alexandre 2020). Chemotaxis has also been reported to enhance infection by several plant pathogens (Matilla and Krell 2018). Several studied examples of virulence-related chemotactic responses include *Pseudomonas syringae* pathovars (Cerna-Vargas *et al*. 2019; Melotto *et al*. 2006), *Ralstonia solanacearum* (Corral *et al*. 2020; Tans-Kersten, Huang and Allen 2001; Yao and Allen 2007) and *Dickeya dadantii* (Antunez-Lamas *et al*. 2009; Rio-Alvarez *et al*. 2015). Similar to animal pathogens, chemotaxis not only enables general attraction of these plant pathogens towards their plant hosts, but it also helps them to localize their preferred sites of infection, such as wounds or open stomata. More generally, other potentially beneficial or opportunistically pathogenic bacterial species in the rhizosphere, such as various *Pseudomonas* and *Bacillus* species use chemotaxis to accumulate towards the nutrient-rich environment around plant roots (Feng *et al*. 2018; Lopez-Farfan *et al*. 2019; Massalha *et al*. 2017). Moreover, it is assumed that chemotaxis towards a specific set of chemicals might enable host plant selection, but how exactly such specific recognition could be achieved remains to be determined.

Similar to these host-microbe interactions in the rhizosphere, motility and chemotaxis might be generally beneficial for the establishment of symbiotic communities and nutrient acquisition by bacteria living in aquatic environments (Fig. 4C). Indeed, active migration and colonization might aid transmission of microbial symbionts between hosts, and the importance of motility has been demonstrated for colonization of several marine animals by their bacterial symbionts (Bright and Bulgheresi 2010; Raina *et al*. 2019). For instance, in the squid-vibrio symbiosis system, *Vibrio fischeri* follows a chitin gradient through ducts and antechambers and actively migrates toward the pores of the light organ in a corkscrew-like motion (Aschtgen *et al*. 2019; Mandel *et al*. 2012). Other squid and cuttlefish species have similar symbiotic consortia composed of bacterial genera like *Roseobacter*, *Pseudoalteromonas*, *Vibrio* and *Shewanella*, which are known for their motility and chemotaxis (Barbieri *et al*. 2001), and chemotaxis might help these bacteria to colonize their hosts and to counter intestinal flow (Wiles *et al*. 2020). Chemotaxis towards planktonic chitin is also thought to be an important component of the ecology of chitin-degrading *Vibrio* species (Erken, Lutz and McDougald 2015). Marine phytoplankton were also shown to secret a range of compounds including dimethylsulfoniopropionate (DMSP), amino acids, sugars and organic acids that attract chemotactic bacterial symbionts (Miller and Belas 2006; Sonnenschein *et al*. 2012; Tout *et al*. 2017). Similarly, the marine macroalga *Ulva mutabilis* (Chlorophyta) releases DMSP to attract chemotactic marine bacteria (Kessler *et al*. 2018).

## CONCLUDING REMARKS

Although motility is among the most studied bacterial behaviors under defined laboratory conditions, its multifaceted importance for the physiology of individual bacteria and microbial communities only recently became appreciated. In this review, we provided an overview of multiple functions of motility, with a primary focus on chemotaxis. As evident from studies of the *E. coli* model, even for the same species chemotaxis might make multiple contributions to physiology, including nutrient acquisition, expansion of the population range, biofilm formation and host colonization. Importantly, these different functions of motility and chemotaxis are not mutually exclusive but context-dependent. Even a single *E. coli* chemoreceptor Tsr can mediate chemotaxis to the preferentially consumed amino acid serine, to bacterial signaling molecules AI-2 and indole, and to animal hormones (Hegde *et al*. 2011; Lopes and Sourjik 2018; Mesibov and Adler 1972; Orr *et al*. 2020; Yang *et al*. 2020). Consequently, knockouts of individual receptors frequently show pleiotropic defects, from reduced growth fitness under conditions where chemotaxis is important to reduced biofilm formation and virulence. Moreover, the deletion of general chemotaxis genes not only impairs chemotaxis but also changes the swimming pattern of bacteria, either making them smooth swimming or tumbly, which can affect surface attachment, collective behaviors or spreading even in absence of specific chemotactic responses. These intertwined effects complicate the mechanistic understanding of the observed impacts of chemotaxis and motility in such complex environments as the rhizosphere or the GI tract, where grains, surfaces or the mucus affect swimming patterns (de Anna *et al*. 2020; Figueroa-Morales *et al*. 2019; Frangipane *et al*. 2019; Galajda *et al*. 2007; Makarchuk *et al*. 2019; Sipos *et al*. 2015; Spagnolie *et al*. 2015), and need to be kept in mind while interpreting such data.

Another outstanding challenge in understanding the physiological and environmental importance of bacterial chemotaxis lies in the characterization of ligand specificity for the many chemotaxis receptors present in different bacterial species. Whereas signaling domains of receptors are highly conserved and can be easily identified bioinformatically, their ligand binding domains and corresponding sensing mechanisms are highly diverse (Ortega, Zhulin and Krell 2017). Although several different approaches to systematically identify ligands for various chemoreceptors have been recently established (Bi *et al*. 2016; Boyeldieu *et al*. 2021; Lehning *et al*. 2017; Luu *et al*. 2019; Matilla, Martin-Mora and Krell 2020), only a tiny fraction of chemoreceptor ligands are currently known and even fewer have an established mode of binding (Ortega, Zhulin and Krell 2017). With the increasing number of characterized ligand-receptor interactions and better understanding of ligand binding by the major structural classes of ligand-binding domains of receptors, computational prediction of ligand specificity should ultimately become possible.
